# Supplementary material for: Mutations in PCYT2 disrupt etherlipid biosynthesis and cause a complex hereditary spastic paraplegia
Source: Brain. 2019 Oct 22;142(11):3382–97. doi: 10.1093/brain/awz291 (PMC6821184; doi:10.1093/brain/awz291)
Supplement: awz291_Supplementary_Data [file awz291_supplementary_data.zip › awz291-Suppl_data/Supplementary_Data4.zip!/Supplementary information 4 - plasma lipidomics.zip!/Plasma lipidomics/Control vs Patient2/Statistical plots and data/OPLS-DA.pdf]

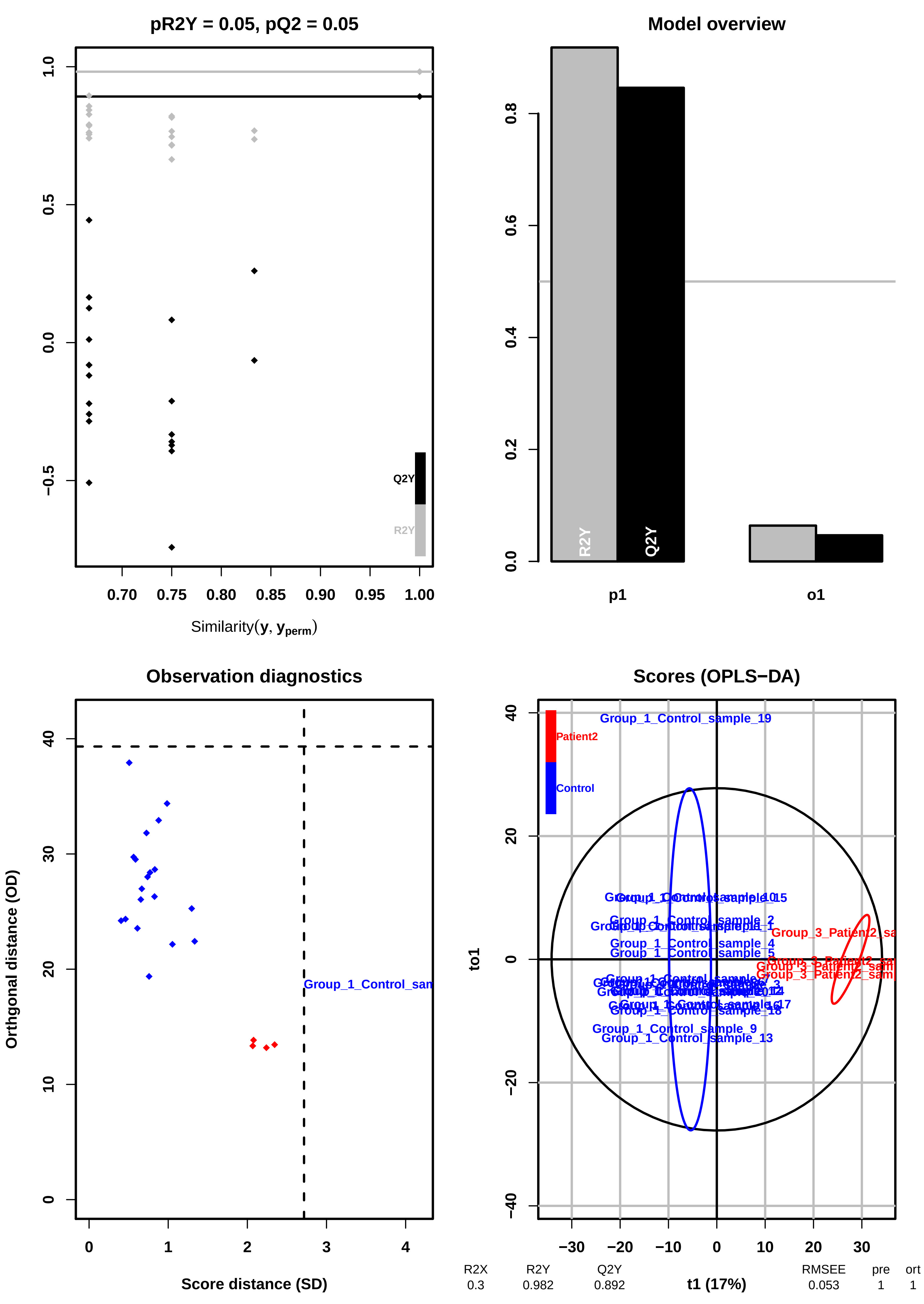

pR2Y = 0.05, pQ2 = 0.05
Q2Y
R2Y
Similarity(y, yperm)
Model overview
R2Y
Q2Y
p1
o1
Observation diagnostics
Orthgonal distance (OD)
Score distance (SD)
Group_1_Control_sam
Scores (OPLS−DA)
Patient2
Control
Group_1_Control_sample_19
Group_1_Control_sample_2
Group_1_Control_sample_4
Group_1_Control_sample_5
Group_1_Control_sample_9
Group_1_Control_sample_13
Group_1_Control_sample_18
Group_3_Patient2_sa
to1
t1 (17%)
R2X 0.3
R2Y 0.982
Q2Y 0.892
RMSEE 0.053
pre 1
ort 1
